# Assessment of Mycotoxin Exposure and Associated Risk in Pregnant Dutch Women: The Human Biomonitoring Approach

**DOI:** 10.3390/toxins16060278

**Published:** 2024-06-18

**Authors:** Hannah P. McKeon, Marloes A. A. Schepens, Annick D. van den Brand, Marjolein H. de Jong, Marleen M. H. J. van Gelder, Marijn L. Hesselink, Marta M. Sopel, Marcel J. B. Mengelers

**Affiliations:** 1 National Institute for Public Health and the Environment (RIVM), 3720 BA Bilthoven, The Netherlandsmarcel.mengelers@rivm.nl (M.J.B.M.); 2 Department for Health Evidence, Radboud University Medical Center, 6525 GA Nijmegen, The Netherlands; marleen.vangelder@radboudumc.nl; 3 Department of Paediatrics, Maastricht University Medical Centre (MUMC+), P. Debyelaan 25, 6229 HX Maastricht, The Netherlands; marijn.hesselink@mumc.nl; 4 School of Nutrition and Translational Research in Metabolism (NUTRIM), Maastricht University, Universiteitssingel 40, 6229 ER Maastricht, The Netherlands; 5 Wageningen Food Safety Research (WSFR), 6708 WB Wageningen, The Netherlands; marta.sopel@wur.nl

**Keywords:** mycotoxins, human biomonitoring, blood, urine, biomarkers, pregnant women, risk assessment

## Abstract

Mycotoxins are toxic secondary metabolites produced by various fungi that can contaminate food crops, which, in turn, may lead to human exposure. Chronic exposure to mycotoxins can cause adverse health effects including reproductive and developmental toxicity. Pregnant women and their foetuses present a vulnerable group for exposure to mycotoxins that can cross the placenta. Human biomonitoring of mycotoxins provides a real-life approach to estimate internal exposure. In this pilot study, 24-h urine samples from 36 pregnant Dutch women were analysed for aflatoxin M1 (AFM1), total deoxynivalenol (DON), de-epoxy-deoxynivalenol (DOM-1), total zearalenone (ZEN), total α-zearalenol (α-ZEL), total β-zearalenol (β-ZEL) and total zearalanone (ZAN), where ‘total’ refers to mycotoxins and their conjugated forms. Serum samples from these women were analysed for fumonisin B1 (FB1) and ochratoxin A (OTA). All samples were measured using liquid chromatography–tandem mass spectrometry (LC-MS/MS). The most prevalent mycotoxins were total DON, total ZEN and OTA, with a detection frequency of 100%. DOM-1, total α-ZEL and total β-ZEL were detected but to a lesser extent, while AFM1, total ZAN and FB1 were undetected. Median concentrations were 4.75 μg total DON/L, 0.0350 μg DOM-1/L, 0.0413 μg total ZEN/L, 0.0379 μg total α-ZEL/L, 0.0189 μg total β-ZEL/L, and 0.121 μg OTA/L. The calculated median concentration for total ZEN and its metabolites was 0.105 μg/L. Based on two separate risk assessment approaches, total DON exposure in this group was considered to be of low concern. Similarly, exposure to total ZEN and its metabolites in this group was of low concern. For OTA, the risk of non-neoplastic effects was of low concern based on exposure in this group, and the risk of neoplastic effects was of low concern in the majority of participants in this group. The findings of this pilot study confirm the presence of mycotoxins in the urine and serum of pregnant Dutch women, with total DON, total ZEN, and OTA most frequently detected. Exposure to all measured mycotoxins was considered to be of low concern in this group, except for exposure to OTA, which was of low concern for the majority of participants. The study’s findings offer valuable insights but should be confirmed using a larger and more diverse sample of the Dutch general population.

## 1. Introduction

Humans are exposed to a variety of mycotoxins mainly through their diet [1]. Mycotoxins are toxic secondary metabolites produced by various species of fungi, predominantly those belonging to the *Aspergillus*, *Fusarium*, *Penicillium, Alternaria* and *Claviceps* genera [2]. Mycotoxins can be present in crops due to humid and warm conditions during growth, storage, transport and processing [3]. Susceptible crops to mycotoxin contamination include cereals, nuts, fruits and vegetables [4]. The Food and Agriculture Organization has estimated that 25% of the world’s food crops are contaminated with mycotoxins [5]. Seasonality and geographical location play a significant role in the occurrence of mycotoxins, as different mycotoxins tend to occur in varied climate conditions. In Central Europe, mycotoxins, including deoxynivalenol (DON), zearalenone (ZEN), fumonisins and ochratoxin A (OTA), are commonly found in food and feed, while in warmer climate zones, aflatoxins are more prevalent [6].

Chronic exposure to certain mycotoxins has been associated with a range of adverse health effects including reproductive and developmental toxicity [7,8,9]. Therefore, pregnant women and their foetuses present a vulnerable group. In experimental animals, aflatoxin B1 (AFB1) affects reproductive and developmental parameters in rodents, already at low doses following short-term exposure [10]. In humans, aflatoxins are known to cross the placenta [11,12], and low birth weight has been associated with AFB1 exposure [13,14,15,16,17,18,19].

ZEN and its metabolites, α-zearalenol (α-ZEL) and β-zearalenol (β-ZEL), have been shown to activate oestrogen receptors in vitro [20], and display uterothrophic activity in vivo, an indicator of oestrogenic potency [21]. In rodents and pigs, ZEN and its metabolites have caused reproductive toxicity [22]. The European Food Safety Authority (EFSA) has set a group tolerable daily intake (TDI) for ZEN and its metabolites based on oestrogenic activity in pigs [22]. Placental transfer of ZEN and its metabolites has been observed in an ex vivo human placental perfusion model [23]. In rats, ZEN and α-ZEL were transferred to the foetus [24] and caused adverse effects [25]. There is limited information on the effects of ZEN in humans; however, exposure has been linked with precocious puberty and breast cancer [21].

In experimental animals exposed to fumonisin B1 (FB1), growth retardation and birth defects, including neural tube defects (NTD) and craniofacial anomalies, have been reported [26], the latter due to apparent interference with folate utilisation [27]. Data on the placental transfer of fumonisins are lacking. In experimental animals, embryo development was shown to be affected by FB1 [28]. In human epidemiological studies, high FB1 exposure was associated with an increased risk of NTDs [29,30,31].

DON and OTA have also exhibited developmental and reproductive toxicity in experimental animals [32,33]. DON has been shown to cross the placenta in sows [34] and in human in vitro and ex vivo models [34]. For OTA, minimal placental transfer was detected in an ex vivo placental perfusion model [35]; however, OTA has been reported in human foetal and placental samples [35,36,37], and transplacental transfer has been demonstrated in experimental animals [38]. Epidemiological research evaluating the effects of DON and OTA on human reproduction is scarce. Exposure to DON was linked with autism in children [39]. In another human study, exposure to OTA was associated with low birth weight [40].

To accurately evaluate the risk of mycotoxin exposure, it is imperative to conduct comprehensive exposure assessments. Human biomonitoring (HBM) can be used to measure internal exposure at the population level to multiple chemicals simultaneously. HBM studies involve the measurement of parent mycotoxins themselves and/or their metabolites in a biological matrix. HBM provides an alternative to dietary exposure assessment, combining occurrence data in food and food consumption data. The dietary exposure assessment approach has been commonly used in risk assessment to date, as it can be used to identify major sources of dietary exposure at a population level. HBM can be a useful tool when investigating all routes of exposure.

Several biomarkers for the most relevant mycotoxins have been validated and implemented to estimate exposure in European populations [41,42,43,44,45,46,47,48]. HBM has already been used to estimate mycotoxin exposure in pregnant women, especially aflatoxins; however, such studies are scarce in Europe. In pregnant Croatian women, DON, its metabolites and OTA were measured in urine [49,50]. Studies carried out in the UK, Norway and Italy similarly used urine as the biological matrix to investigate single mycotoxin exposure in pregnant women [51,52,53]. In general, urine is a commonly used matrix in HBM as it is non-invasive and relatively convenient to collect. Urine reflects the body’s excretion of chemicals, providing information on recent internal exposure. For chemicals that are quickly renally cleared from the body (e.g., aflatoxins and DON and ZEN), 24-h urine can capture daily exposure. Alternatively, FB1 and OTA are often measured in serum rather than urine to reflect steady-state exposure. For FB1, this is due to its low bioavailability. The major fraction of FB1 is not absorbed but is excreted unchanged in the faeces, with maximally 1% excreted via urine [54,55]. Urine has been used to estimate exposure to FB1 in humans [56]; however, serum, plasma or hair are preferred biological matrixes to measure long-term exposure [57]. For OTA, serum is commonly used to measure exposure due to its long half-life and high protein binding affinity [58,59,60]. Only the non-protein-bound fraction of OTA will pass through glomerular filtration in the kidneys, resulting in low urinary excretion.

In this pilot study, urine and serum samples from pregnant Dutch women in their first trimester were analysed for the presence of several mycotoxins at a single timepoint. This is the first study to measure multiple mycotoxins in pregnant Dutch women using the HBM approach. Mycotoxins were selected based on their exposure, reproductive and developmental effects, possibility of placental transfer and availability of validated exposure biomarkers in urine or blood. To this end, AFM1 (a monohydroxylated metabolite of AFB1), total DON, de-epoxy-DON (DOM-1) (a non-toxic metabolite of DON), total ZEN, total α-ZEL, total β-ZEL and total zearalanone (ZAN), where ‘total’ refers to mycotoxins and their conjugated forms, were measured in 24-h urine, while OTA and FB1 were measured in serum using liquid chromatography–tandem mass spectrometry (LC-MS/MS). A risk assessment was conducted to investigate the safety of exposure to the measured mycotoxins in this group of participants.

## 2. Results and Discussion

This study measured the concentration of several mycotoxins in 24-h urine and serum samples of 36 pregnant Dutch women using LC-MS/MS. The mean age of the participants was 31 ± 3 years (range: 28–39 years), and the mean body weight was 65 ± 14 kg (range: 49–112 kg). The mean 24-h urine volume of the participants was 2.1 ± 0.8 L (range: 0.8–5.1 L). One participant had an outlying urinary volume of 5.1 L. This value was checked and found to be indeed entered into the form. In addition, this participants’ urinary mycotoxin concentrations were in line with those of the other participants. Therefore, this participant was included in the present research.

The results of the measured mycotoxins are described in Section 2.1. The results were compared to the results of other European HBM studies in which the same mycotoxins were analysed in (non-)pregnant women (Section 2.2). Subsequently, a risk assessment was conducted for the detected mycotoxins (Section 2.3). A mixture risk assessment was not performed as the critical effects of the investigated mycotoxins differed. Finally, the limitations of this study and future recommendations are described in Section 2.4.

### 2.1. Mycotoxin Concentrations

Urine samples were analysed for AFM1, total DON, DOM-1, total ZEN, total α-ZEL, total β-ZEL and total ZAN. Serum samples were analysed for FB1 and OTA. The results of the mycotoxin analysis are summarised in Table 1. For total DON, total ZEN and OTA, 100% of the participants’ samples were analysed above the limit of quantification (LOQ). DOM-1, total α-ZEL and total β-ZEL were detected but to a lesser extent. AFM1, total ZAN and FB1 were not detected in any of the samples. Considering that the limit of detection (LOD) values for AFM1 and FB1 were quite low, and therefore sufficiently sensitive, it was acceptable to conclude that their exposure was of low concern in this group. Median mycotoxin concentrations (not adjusted for dilution) were 4.75 μg total DON/L, 0.0350 μg DOM-1/L, 0.0413 μg total ZEN/L, 0.0379 μg total α-ZEL/L, 0.0189 μg total β-ZEL/L and 0.121 μg OTA/L. The calculated median concentration for total ZEN and its metabolites was 0.105 μg/L.

### 2.2. Comparison with Other European Groups

Table 2 shows a summary of the results from previous European HBM studies in (non-)pregnant women. HBM studies investigating the mycotoxins which only report concentrations for the total group of participants, not stratified by sex, were not used for comparison with the results of the present study since we were particularly interested in exposure in women of childbearing age. In addition, mycotoxin excretion profiles may differ depending on sex. Such differences were reported for DON and its conjugated metabolites in a study by Vidal et al. [44], whereby women excreted significantly more total DON, DON-3-glucuronide and DON-15-glucuronide than men over a 24-h period. Concentrations of (total) DON, total ZEN and its metabolites and OTA were investigated in the studies presented. HBM studies reporting concentrations of AFM1 and FB1 in European (non-)pregnant women were not identified.

All of the identified studies measured OTA in urine, specifically in first morning void (FMV) urine. Therefore, such results could not be directly compared to those of the present study, since OTA was measured in serum. Serum is the generally accepted biological matrix for internal exposure estimates of OTA [60], and research has demonstrated that OTA concentrations excreted in urine are considerably lower than circulating OTA concentrations in blood [61]. Nevertheless, it is interesting to note that OTA was frequently detected in urine of Croatian pregnant women (10–100%) [49,50].

The reported detection frequency of (total) DON in urine samples of pregnant women from Croatia, the UK and Norway were in line with that reported in the present study [49,51,52,53]. The frequency reported in pregnant Italian women was approximately two-fold lower [53], which could be explained by country-specific dietary habits or differences in method sensitivity. The LOD used in the Italian study (LOD = 0.25 μg/L) was higher than that used in the Norwegian (LOD = 0.005 μg/L), British (LOD = 0.12 μg/L) and present study (LOD = 0.07 μg/L).

Urinary concentrations of total DON reported in pregnant Croatian women were exceptionally high, with a median value of 48.7 μg/L [49], approximately 10-fold higher than that reported in the present study. The authors reported that the high exposure was likely due to the heavy rainfall which occurred in the previous season, resulting in high concentrations in cereal crops [49]. Median concentrations of total DON in pregnant women from the UK, Norway and Italy were reported by Brera et al. [53]. The median total DON concentration reported in pregnant Norwegian women was very similar to that in the present study, whereas the median total DON concentrations reported in pregnant women from the UK and Italy were three-fold higher and two-fold lower, respectively. In the mentioned studies investigating total DON, FMV urine was used as the sampling method and the concentrations were reported as measured values and dilution adjusted values (expressed per gram of creatinine). In the present study, concentrations were not adjusted for dilution; however, 24-h urine was used, which reflects variations in exposure throughout the day, providing a comprehensive view of daily exposure. Overall, it is difficult to compare results generated from different sampling methods. FMV is typically more concentrated than 24-h urine; however, studies have reported a good correlation between the two [62]. In the future, it would be beneficial to also adjust 24-h urine concentrations for creatinine to facilitate comparison with the findings of other studies.

It is also interesting to compare mycotoxin exposure in pregnant women to non-pregnant women from the general population since there may be differences in dietary intake. HBM of mycotoxins in the Dutch general population has not yet been performed. In Germany, total DON was analysed in 24-h urine samples of young women [46]. The reported detection frequency and median concentration of total DON were in line with that reported in the present study, indicating that exposure in pregnant women in the Netherlands may be similar to that in non-pregnant women of similar age in neighbouring countries.

Total ZEN, total α-ZEL and total β-ZEL were analysed in spot urine samples of German young women [63], and the reported detection frequency and median concentrations were slightly higher but generally in line with that of the present study. Small differences in exposure could be due to varied dietary intake patterns or possibly the time of sampling. To note again, it is a challenge to draw comparisons between results generated from different sampling methods.

**Table 2:** Summary of results from other HBM studies investigating (total) DON, total ZEN and its metabolites and OTA exposure in (non-)pregnant women in Europe.

Country	Collection Year	Study Population	Biological Matrix	Mycotoxin	Positive %	Median (μg/L)	Range (μg/L)	Method Sensitivity (μg/L)	Reference
**The Netherlands**	2020–2022	36 pregnant women	24-h urine Serum	total DON ^1^ total ZEN ^2^ total α-ZEL ^2^ total β-ZEL ^2^ OTA	100 100 75 50 100	4.75 0.0413 0.0379 0.0189 0.121	1.26–21.6 0.01–0.138 <LOD-0.115 <LOD-0.058 0.0586–2.26	LOD = 0.07 LOD = 0.005 LOD = 0.025 LOD = 0.025 LOD = 0.05	Present study
**Croatia**	2011	40 pregnant women	FMV urine	DON DON15GlcA DON3GlcA total DON ^3^ OTA	76 98 83 - 10	6.7 55.2 10.0 48.7 <LOQ	<LOD-275 <LOD-1237 <LOD-298 4.8–1238 -	LOD = 4 LOD = 3 LOD = 6 - LOD = 0.05	[49]
**Croatia**	2011	40 pregnant women	FMV urine	OTA OTα	78 100	0.02 1.18	<LOD-1.11 0.11–7.57	LOD = 0.019 LOD = 0.016	[50]
**UK**	2008–2009	85 pregnant women	24-h urine	total DON	100	-	0.5–117 ^2^	-	[51]
**UK**	2014	42 pregnant women	FMV urine	total DON	88	14.3 ^4^	-	LOD = 0.12	[52,53]
**Italy**	2014	42 pregnant women	FMV urine	total DON	43	1.96 ^4^	^-^	LOD = 0.25	[53]
**Norway**	2014	40 pregnant women	FMV urine	total DON	100	5.29 ^4^	^-^	LOD = 0.005	[53]
**Germany**	1996–2021	180 females	24-h urine	total DON	99	3.54	<LLOQ-26.4	LLOQ = 0.3	[46]
**Germany**	2013–2014	30 females	FMV urine	total ZEN total α-ZEL total β-ZEL	100 100 100	0.07 0.12 0.03	0.04–0.23 0.09–0.45 0.01–0.20	LOD = 0.01 LOD = 0.01 LOD = 0.01	[63]

DON: deoxynivalenol; DON15GlcA: DON-15-glucuronide; DON3GlcA: DON-3-glucuronide; FMV: first morning void; HBM: human biomonitoring; LOD: limit of detection; LOQ: limit of quantification; LLOQ: Lower LOQ; OTA: ochratoxin A; OTα: ochratoxin α; ZEN: zearalenone; α-ZEL: α-zearalenol; β-ZEL: β-zearalenol; -: not applicable or reported. ^1^ Total DON refers to DON and its glucuronides. ^2^ Total ZEN, total α-ZEL, total β-ZEL refers to the mycotoxins and their glucuronides and sulphates. ^3^ Summed value of DON, DON15GlcA and DON3GlcA taking molecular weight into account. ^4^ Reported as μg/gram creatinine.

### 2.3. Risk Assessment

#### 2.3.1. DON

Two approaches were used to assess the risk of total DON measured in participants’ urine samples. Firstly, urinary concentrations were directly compared to the HBM-GV for total DON using the hazard quotient (HQ) approach. This approach is commonly used in risk assessment to estimate the potential risk of adverse effects to organisms exposed to specific chemicals. An HQ value of 1 indicates that exposure equals the health-based guidance value. When the HQ ≤ 1, no appreciable adverse health effect is expected (low concern). In contrast, when the HQ > 1, adverse health effects cannot be excluded (potential concern).

An HBM-GV of 23 μg/L for total DON (DON and its glucuronides) in urine was derived within the European Human Biomonitoring Initiative (HBM4EU), using the group-TDI set by EFSA in 2017 as starting point for the derivation [64]. The group-TDI was set at 1 μg/kg bw for the sum of DON, 3-acetyl-DON, 15-acetyl-DON and DON-3-glucoside based on reduced body weight gain in mice [32].

**Figure 1:**
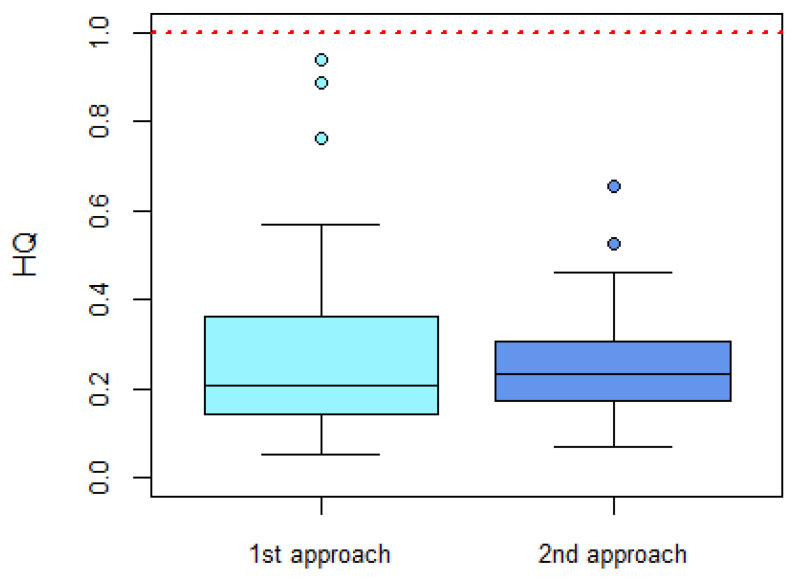
Hazard quotients (HQ) for DON exposure in participants (*n* = 36) calculated using two different approaches. In the ‘1st approach’, urinary total DON concentrations were directly compared to the HBM-GV. In the ‘2nd approach’, calculated EDIs were compared to the group-TDI for DON and its conjugated forms. The median is the black line within the box. The bottom and top of the box represent the 25th and 75th percentiles, respectively. The whiskers represent the min and max values and the outliers are depicted by the single points. The dashed red line indicates where exposure equals the health-based guidance value.

All participants had total DON concentrations below the HBM-GV. Therefore, all calculated HQs were below 1, indicating that the level of exposure in this group was considered to be of low concern (Figure 1).

Secondly, urinary total DON concentrations were used to calculate the estimated daily intakes (EDI) of all participants using reverse dosimetry (see Section 4) and are displayed in Table 3.

As a second approach, EDIs were compared to the group-TDI of 1 μg/kg bw for DON and its conjugated forms [32]. EDIs calculated for all participants were below the group-TDI, and consequently, all HQs were below 1 (Figure 1), indicating that the level of exposure was of low concern.

The two risk assessment approaches yielded similar results. Slight differences between the findings of both approaches are apparent since the first approach directly compares the urinary total DON concentrations with the HBM-GV, not taking into account specific participant details (e.g., body weight or urinary volume), while the second approach back-calculates the EDIs for each participant, taking into account their individual body weight and 24-h urinary volume. Overall, both approaches introduce uncertainty, and choosing an approach depends on the objective of the study. Selecting the first approach is a practical and simple approach to conduct a risk assessment, especially with a large sample size, while choosing the second approach takes into account specific participant parameters and can be used to assess risks when a HBM-GV is unavailable and the risk needs to be assessed using an external health-based guidance value (e.g., TDI).

Other studies have conducted risk assessments in European pregnant women using EDI calculations. In a group of pregnant women from the UK, Wells et al. [52] reported that 25% of the participants exceeded the group-TDI. Not surprisingly, in the study by Šarkanj et al. [49], which reported a 10-fold higher median urinary total DON concentration than that reported in this study, 48% of pregnant Croatian women were estimated to exceed the group-TDI based on urinary concentrations of total DON.

Previously, EDIs for DON and other mycotoxins were calculated for the Dutch general population (aged 7–69 years) using the dietary exposure assessment approach [65]. At the median and 95th percentile, upper bound EDIs were 0.124 and 0.354 μg DON/kg bw/day, respectively, and Sprong et al. [65] reported exposure to be of low concern. Median and 95th percentile DON EDIs are slightly lower but similar to those calculated in the present study, indicating that exposure may not differ greatly between sub-populations in the Netherlands and that exposure may be similar to that of previous years.

#### 2.3.2. ZEN

Currently, no HBM-GV or other internal health-based guidance value has been derived for ZEN and its metabolites in urine. Calculated urinary concentrations of total ZEN and its metabolites (total α-ZEL, total β-ZEL and total ZAN) were therefore used to calculate the EDI for each participant using reverse dosimetry (see Section 4). The calculated EDIs are displayed in Table 4.

An important parameter in the EDI calculation is the urinary excretion fraction (FUE). Due to the limited availability of toxicokinetic data for ZEN and its metabolites in humans, an FUE range of 0.094–0.368 (9.4–36.8%%) was applied in our calculations based on published literature. In a human study with one male volunteer, the excretion of ZEN and its metabolites, α-ZEL, and β-ZEL, was analysed in urine following a single oral dose of 100 mg ZEN [66]. Based on the concentration data, it was estimated that 10–20% of the administered dose was excreted within 24 h [67]. A more recent study involving one healthy male exposed to a daily dose of 10 µg ZEN reported a mean urinary excretion percentage of 9.4% (range: 7.0–13.2%) for ZEN (including its glucuronide forms) over 24 h [68]. However, α-ZEL and β-ZEL were not covered. In a piglet study, a mean urinary excretion percentage of 36.8% (including 28.4% as ZEN and 8.3% as α-ZEL) was reported [69], and this has been applied in several HBM studies due to limited human data [45,70,71,72,73]. By applying physiologically based pharmacokinetic modelling, the mean urinary excretion percentage of ZEN and its metabolites was estimated to be 15.8–18.1% in humans [74].

**Figure 2:**
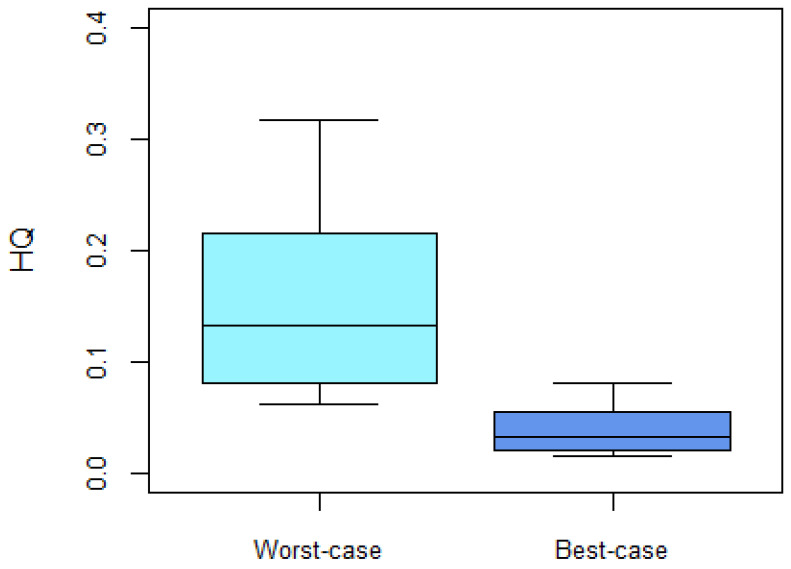
Hazard quotients (HQ) for ‘Worst-case’ (FUE = 0.094) and ‘Best-case’ (FUE = 0.368) ZEN exposure in participants (*n* = 36). The median is the black line within the box. The bottom and top of the box represent the 25th and 75th percentiles, respectively. The whiskers represent the min and max values.

Using the HQ approach, the calculated EDIs were compared to the group-TDI for ZEN and its metabolites, which has been set by EFSA at 0.25 μg/kg bw [21]. In both the worst- and best-case scenarios, all participants’ EDIs were well below the group-TDI. Therefore, all calculated HQs were below 1, indicating that the level of exposure in this group was of low concern (Figure 2). To note, no HQ cut-off value of 1 is given in the figure since participants’ HQ values were much lower.

Sprong et al. [65] previously calculated EDIs for ZEN, α-ZEL, and β-ZEL for the Dutch general population (aged 7–69 years) using the dietary exposure assessment approach [64]. At the median and 95th percentile of the group, the calculated upper bound EDIs were 0.0091 and 0.0272 µg ZEN/kg bw/day, 0.00006 and 0.0053 µg α-ZEL/kg bw/day, and 0.00006 and 0.0053 µg β-ZEL/kg bw/day. Using these data and correcting for molecular weight, the EDIs for the sum of ZEN and its metabolites were calculated to be 0.0092 and 0.0377 µg/kg bw/day for the upper-bound median and 95th percentile, respectively, which are values similar to the best-case scenario EDI calculations in the present study. This indicates that exposure to ZEN and its metabolites may not differ greatly between this sub-population of pregnant Dutch women and the Dutch general population.

#### 2.3.3. OTA

For OTA, an HBM-GV or other internal health-based guidance value has yet to be established. Serum OTA concentrations were used to calculate the EDIs of participants using reverse dosimetry (see Section 4), which are displayed in Table 5.

Due to uncertainty regarding the mode of action for OTA-induced kidney carcinogenicity, no external health-based guidance value for OTA is currently applicable. EFSA derived a BMDL_10_ of 4.73 μg/kg bw/day for kidney lesions observed in pigs (non-neoplastic effects) and a BMDL_10_ of 14.5 μg/kg bw/day for kidney tumours seen in rats (neoplastic effects) [33]. The calculated EDIs were compared to the BMDL_10_ values using an adapted HQ calculation incorporating the margin of exposure (MOE) approach (see Section 4). The MOE is the ratio of the point of departure (PoD), in our case, the BMDL_10_ value, to the external exposure, thereby expressing the margin between the dose at which an adverse effect can be observed and the exposure. According to EFSA [33], for OTA related non-neoplastic effects, an MOE ≥ 200 is considered to be of low concern, while for neoplastic effects, an MOE ≥ 10,000 is considered to be of low concern.

**Figure 3:**
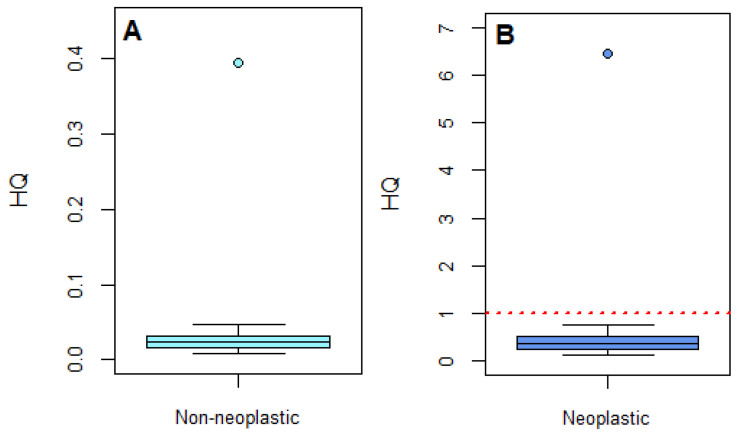
Hazard quotients (HQ) for OTA exposure in participants (*n* = 36) in relation to ‘Non-neoplastic’ (**A**) and ‘Neoplastic’ effects (**B**). The median is the black line within the box. The bottom and top of the box represent the 25th and 75th percentiles, respectively. The whiskers represent the min and max values, and the outliers are depicted by the single points. For ‘Neoplastic’ effects, the dashed red line indicates where the margin of exposure equals a value of 10,000.

With regard to non-neoplastic effects, all participants had MOEs ≥ 200. Therefore, all calculated HQs were below 1, and the risk for non-neoplastic effects resulting from OTA exposure in this group was considered to be of low concern (Figure 3A). To note, no HQ cut-off value of 1 is given in Figure 3A since participants HQ values were much lower. For neoplastic effects, 97% (35/36) of the participants had MOEs ≥ 10,000 (Figure 3B). One participant had an MOE < 10,000 (1549) and thus an HQ value above 1, which indicates a possible health concern. This individual was a major outlier in the group with a calculated EDI value of 9.36 ng/kg bw/day, which was approximately 20-fold higher than the median value. The large differences between this individual and the rest of the group are likely due to deviations in dietary intake and/or additional occupational exposure. However, this consideration could not be confirmed since the dietary intake patterns of the participants were not known. In future HBM studies, the collection of dietary records and other participant-specific information (e.g., occupation) would be beneficial for determining the route of exposure.

Previously, Sprong et al. [65] concluded that health risks could not be excluded for OTA based on dietary exposure estimations in the Dutch general population (aged 7–69 years). The reported upper-bound median and 95th percentile EDIs were 7.1 and 19.4 ng OTA/kg bw/day, respectively, which are approximately 13- and 19-fold higher than those calculated in the present study, respectively. Sprong et al. [65] reported that coffee consumption was an important contributor to OTA exposure in adults, accounting for 73% of the exposure. Exposure differences within the Dutch population could be explained by the specific dietary recommendations for pregnant women. Pregnant women are advised to limit their intake of caffeine [75] as it may cause adverse outcomes in the developing foetus. Coffee is a major source of caffeine and is also commonly contaminated with OTA [76], so by avoiding this source of OTA, pregnant women could be less exposed.

### 2.4. Limitations and Future Recommendations

This pilot study is a valuable first step in exploring the risks associated with mycotoxin exposure in a small group of 36 pregnant Dutch women. The women included in this study were part of the JOZO project, which is a larger study investigating the association between iodine intake and mother and child health. As a result, the sample collection locations were limited to two participating hospitals in Nijmegen and Maastricht. The relatively small sample size and lack of geographical diversity limits the generalisability of the findings to a broader population. However, it is important to highlight that this study was designed as a pilot, which by nature focuses on a small scale to assess the feasibility of the methods and explore initial trends. Pilot studies are not intended to be representative of an entire population but rather to provide valuable insights and inform the design of a larger and more comprehensive study. In our case, the aim was to measure multiple mycotoxins in pregnant Dutch women using the HBM approach, which required meticulous analysis of urine and serum samples. We believe that despite the limited sample size, our pilot study provides valuable preliminary data on the presence of mycotoxins in this group. Moving forward, we aim to expand our study to include a larger and more diverse group of the Dutch general population.

Given the high detection frequency of total DON, total ZEN and OTA in this group of participants, future HBM of these mycotoxins should be conducted in the Netherlands to further monitor the associated risks. From the findings in the preliminary risk assessment, focus could be placed on the HBM of OTA since one participant was above the cut-off value for risk of neoplastic effects. In relation to future biomonitoring of mycotoxins in urine, adjusting for dilution could be considered to allow comparison with concentrations in other studies.

In general, the interpretation of HBM data is hindered by the lack of mycotoxin specific toxicokinetic data. Knowledge of excretion patterns is critical for accurate and realistic EDI calculations based on urinary mycotoxin concentrations. The toxicokinetics of some mycotoxins, such as DON and OTA, have been studied in humans more extensively than others. For ZEN and its metabolites, there is a lack of quantitative excretion data in humans leading to large uncertainties in EDI calculations. Due to the lack of excretion data for ZEN, an FUE range was applied in our EDI calculations, which included human, animal and modelled data with high uncertainty (see Section 4). Additionally, ZEN and its metabolites have a relatively long half-life compared to other mycotoxins such as DON, which makes it difficult to completely capture daily ZEN exposure using 24-h urine samples. This data gap could be solved by means of a duplicate diet study, whereby the exact ingested dose is known and excretion is captured over a particular timeframe. With such data from a more controlled human study, the risk of exposure could be better understood and assessed.

## 3. Conclusions

This study has provided novel data on mycotoxin concentrations in urine and serum samples obtained from 36 Dutch women in their first trimester of pregnancy. The most frequently detected mycotoxins were total DON, total ZEN and OTA. DOM-1, total α-ZEL and total β-ZEL were detected but to a lesser extent, while AFM1, total ZAN and FB1 were undetected. The associated risk of exposure to the most frequently detected mycotoxins, total DON, total ZEN and OTA, was then investigated. Exposure to total DON and total ZEN and its metabolites in this group was considered to be of low concern. With regard to OTA exposure in this group, the risk of non-neoplastic effects was of low concern. For the risk of neoplastic effects, OTA exposure in the majority of this group was of low concern, except for one participant, whereby a risk could not be excluded. The findings presented in this study provide valuable insights into the efficacy of HBM in assessing the risk associated with mycotoxin exposure. Considering that the present investigation was carried out on a small group of women from two particular locations in the Netherlands, the findings should be considered preliminary and used for the design of a larger and more comprehensive HBM study. Moreover, controlled human studies are required to better understand the toxicokinetics of ZEN and its metabolites in the body, and with this, the risk of exposure can be better understood and assessed.

## 4. Materials and Methods

### 4.1. Study Participants

Twenty-four-hour urine samples and serum samples were obtained from 36 healthy Dutch women in their first trimester of pregnancy. The participants collected urine over a 24-h period of time, and before sample analysis, the samples were homogenised. The sample collection period was between October 2020 and December 2022. The sample collection period had to be extended due to the COVID-19 pandemic. Participants had a mean age of 31 ± 3 years and a mean body weight of 65 ± 14 kg. The participants were part of the JOZO project, which is a large study among pregnant women throughout the Netherlands investigating the associations between iodine intake and mother and child health. Written consent was provided by the participants for the use of their data and samples. The study was approved by the local Medical Ethical Committee of Maastricht University Medical Center+ (MUMC+) and the METC Oost Nederland. Data and sample collection took place at two locations, MUMC+ (23 participants) (Maastricht) and Radboud University Medical Center (Nijmegen) (13 participants). Overall, a total of 36 urine and serum samples were subject to analysis. Urine and serum samples were stored at −80 °C until analysis. In addition, the participants filled out a web-based general questionnaire which was designed for the JOZO study.

### 4.2. Chemicals

Analytical standards of AFM1, ^13^C_17_ AFLA M1, OTA, ^13^C_20_ OTA, FB1, ^13^C_34_ FB1, DON, ^13^C_15_ DON, DOM-1, ZEN, ZAN, α-ZEL, β-ZEL and ^13^C_18_ ZEN were obtained from Biopure™, while α-ZEL-d_7_ and β-ZEL-d_7_ were obtained from TRC. Immunoaffinity columns EASI-EXTRACT^®^ Aflatoxin M1, Ochraprep, Fumoniprep and Easi-Extract Zearalenone were obtained from R-Biopharm, and DONTEST IAC columns were purchased from Vicam. β-Glucuronidase from *Escherichia coli* type IX-A (1 MU/g) and β-Glucuronidase/Arylsulfatase from *Helix pomatia* (10.8 U/mL ß-Glucuronidase and 25 U/mL Arylsulfatase) were purchased from Sigma-Aldrich. Potassium dihydrogen phosphate (K_2_HPO_4_), ammonium formate, formic acid, acetic acid and sodium acetate anhydrous were purchased from Merck. Acetonitrile and methanol were obtained from Biosolve. Potassium phosphate dibasic (KH_2_PO_4_), phosphate-buffered saline sachet containing powder pH 7.4 for preparing 1 L solution and ammonium formate >99% were purchased from Sigma-Aldrich, and formic acid 98–100% was obtained from LiChropur^®^.

### 4.3. Sample Preparation and Analysis

#### 4.3.1. Urine

For AFM1, an aliquot of 5 mL urine was mixed with 2 mL of potassium phosphate buffer pH 6.8. A deconjugation step was performed overnight at 37 °C before the analysis of DON, DOM-1, ZEN, α-ZEL, β-ZEL and ZAN, so that free and conjugated forms could be analysed. When free and conjugated forms of the mycotoxins were analysed, this is referred to as ‘total’ throughout the manuscript. For total DON and DOM-1, a deconjugation step was performed using 1 mL urine diluted with 2 mL potassium phosphate buffer pH 6.8 and 240 μL deconjugation enzyme β-Glucuronidase derived from *Escherichia coli*, which has the ability to deconjugate urinary glucuronides. For total ZEN, total α-ZEL, total β-ZEL and total ZAN, deconjugation was performed using 4 mL urine diluted with 4 mL acetate buffer pH 4.5 and 40 µL β-Glucuronidase/Arylsulfatase derived from *Helix pomatia*, which have the ability to deconjugate both urinary glucuronides and sulphates.

The analytes of AFM1, total DON, DOM-1, total ZEN, total α-ZEL, total β-ZEL and total ZAN were extracted and cleaned on immunoaffinity columns (IAC) dedicated to the specific mycotoxin/metabolite and then analysed by LC-MS/MS.

#### 4.3.2. Serum

For FB1, 500 µL of serum was diluted with 3.5 mL of potassium phosphate buffer pH 7.4. For OTA, 400 µL of serum was diluted with 500 µL acetonitrile, and the sample was vortexed to precipitate the proteins. The sample was then centrifuged, and the supernatant was transferred and diluted with 9 mL of potassium phosphate buffer pH 7.4.

The analytes were extracted on IAC columns dedicated to FB1 or OTA and then analysed by LC-MS/MS.

#### 4.3.3. LC-MS/MS

Two different LC-MS/MS systems were used: (1) Waters Xevo TQS with ESI interface; and (2) Sciex QTRAP 6500 with ESI interface. Specifications of the LC-MS/MS including the manufacturer and model, and also the applied analytical columns, mobile phases, gradients, MS/MS parameters and m/z ions monitored, are provided in Table S1 of the Supplementary Materials. Quantification was carried out by external calibration of standards in solvent after normalisation of the response to the isotopically labelled internal standard (IS) which were ^13^C_17_ AFLA M1, ^13^C_15_ DON, ^13^C_18_ ZEN, α-ZEL-d_7_, β-ZEL-d_7_, ^13^C_34_ FB1 and ^13^C_20_ OTA. The IS were added before the sample extraction on IAC columns for AFM1, OTA and FB1, and to the reaction mixture for urine incubation with enzymes for DON and ZEN. For DOM-1 and ZAN, the corresponding IS were not available; therefore, the quantification was performed using external calibration of standards in solvent. The recovery, repeatability, LOD and LOQ values of AFM1, tDON, DOM-1, ZEN, α-ZEL, β-ZEL and ZAN in urine and of FB1 and OTA in serum are provided in Table 6.

### 4.4. External Exposure Estimate Model

#### 4.4.1. DON and ZEN

Urinary concentrations of total DON and total ZEN and its metabolites (total α-ZEL, total β-ZEL and total ZAN) measured in this study were used to estimate the EDI of each participant by applying a version of the formula derived by Solfrizzo et al. [45]. The calculation is expressed as follows:

$$EDI=\frac{C\times V}{BW\times FUE}$$

where:

EDI = estimated daily intake (µg/kg bw/day);
C = urinary mycotoxin concentration (µg/L);
V = 24-h urinary volume of each participant (L)
BW = body weight measured for each participant (kg);
FUE = mean urinary excretion fraction of DON ^1^ and ZEN ^2^ (0 < FUE < 1).
^1^ The mean FUE for DON used in this study was 0.69 calculated with 24-h urine from 20 healthy adults (11 women and 9 men) [64,77].
^2^ Due to the limited availability of toxicokinetic data for ZEN and its metabolites in humans, an FUE range of 0.094–0.368 (9.4–36.8%) was applied to take all published urinary excretion data into account [66,67,68,69,74].

#### 4.4.2. OTA

Serum concentrations measured in this study were used to estimate the EDI of OTA for each participant using parameters derived by Studer-Rohr et al. [78]. They calculated total body clearance to be 0.0935 mL/min (134.6 mL/day) from the plasma concentration–time curve in humans. The fraction absorbed for OTA was calculated to be 0.5 by Studer-Rohr [79] and then as 0.8 more recently by Studer-Rohr et al. [78]. In our calculations, the fraction absorbed of 0.5 was used as the worst-case scenario. The calculation is expressed as follows:

$$EDI=\frac{CLtot\times Cav.ss}{BW\times F}$$

where:

EDI = estimated daily intake (ng/kg bw/day);
CLtot = total body clearance (mL/day);
Cav.ss = average OTA concentration in plasma at steady-state (ng/mL);
BW = body weight measured for each participant (kg);
F = fraction absorbed (0 < F <1).

### 4.5. Risk Assessment

HQ is an approach used in risk assessment to estimate the potential risk of adverse effects to organisms exposed to specific chemicals. In relation to non-cancer effects, the HQ can be expressed as follows:

$$HQ=\frac{\mathrm{Human}\;\mathrm{exposure}\;\mathrm{level}\;}{\mathrm{Health}-\mathrm{based}\;\mathrm{guidance}\;\mathrm{value}}$$

The HQ provides a relative measure of the potential risk associated with the exposure to the chemical. When the HQ ≤ 1, no appreciable adverse health effect is expected. In contrast, when the HQ > 1, adverse health effects cannot be excluded. This approach can be used to compare chemical concentrations to internal health-based guidance values (e.g., HBM-GV) or to compare EDIs to external health-based guidance values (e.g., TDI).

In cases where no health-based guidance value is available for a chemical, the HQ equation must be adapted. For instance, for chemicals which are genotoxic carcinogens, a health-based guidance value cannot be derived, as no level of exposure is considered safe (e.g., AFB1). For OTA, due to uncertainty regarding the mode of action for kidney carcinogenicity, no health-based guidance value has been derived. Using the MOE approach, EDIs can be compared to a selected PoD (e.g., BMDL_10_). For OTA and its associated non-neoplastic effects, EFSA reported that an MOE ≥ 200 is considered to be of low health concern, while for neoplastic effects, an MOE ≥ 10,000 is considered to be of low health concern [33]. For non-neoplastic effects, the HQ can be rewritten as follows:

$$HQ=\frac{200}{MOE}$$

For neoplastic effects, the HQ can be rewritten as follows:

$$HQ=\frac{10,000}{MOE}$$

The MOE is the ratio of the PoD to the external exposure, thereby expressing the margin between the dose at which an adverse effect can be observed and the exposure. EFSA has recommend that a BMDL should be used as the PoD to obtain an MOE [80]. The MOE can be calculated as follows:

$$MOE=\frac{PoD}{\mathrm{Human}\;\mathrm{exposure}\;\mathrm{level}}$$

In our case, the human exposure level is equal to the EDI.

## Figures and Tables

**Table 1:** Geometric mean, average, median and 95th percentile mycotoxin concentrations in urine and serum samples of participants (*n* = 36).

Mycotoxin	Geometric Mean ^1^	Average ± SD	Median	95th Percentile	Range	% >LOD/LOQ ^6^
Urine (μg/L)						
**AFM1**	<LOD	<LOD	<LOD	<LOD	-	0/0
**total DON ^2^**	5.27	6.67 ± 5.02	4.75	18.3	1.26–21.6	100/100
**DOM-1**	0.0419	0.0457 ± 0.0237	0.0350	0.0925	<LOD-0.130	19/0
**total ZEN ^3^**	0.0371	0.0458 ± 0.0309	0.0413	0.106	0.01–0.138	100/100
**total α-ZEL ^3^**	0.0326	0.0397 ± 0.0253	0.0379	0.0821	<LOD-0.115	75/22
**total β-ZEL ^3^**	0.0218	0.0260 ± 0.0156	0.0189	0.0569	<LOD-0.058	50/11
**total ZAN ^3^**	<LOD	<LOD	<LOD	<LOD	-	0/0
**∑ZEN ^4^**	0.101	0.118 ± 0.0677	0.105	0.239	0.0417–0.3115	-
Serum (μg/L)						
**FB1**	<LOD	<LOD	<LOD	<LOD	-	0/0
**OTA**	0.132	0.192 ± 0.360 ^5^	0.121	0.291	0.0586–2.26	100/100

AFM1: aflatoxin M1; DOM-1: de-epoxy-DON; DON: deoxynivalenol; FB1: fumonisin B1; LOD: limit of detection; LOQ: limit of quantification; OTA: ochratoxin A; SD: standard deviation; ZAN: zearalanone; ZEN: zearalenone; α-ZEL: α-zearalenol; β-ZEL: β-zearalenol; -: not applicable. ^1^ When calculating means and percentiles, concentrations below LOD were set to half the value of the LOD. ^2^ Total DON refers to DON and its glucuronides. ^3^ Total ZEN, total α-ZEL, total β-ZEL and total ZAN refers to the mycotoxins and their glucuronides and sulphates. ^4^ The sum of total ZEN, total α-ZEL, total β-ZEL and total ZAN after correction for molecular weight (mW) (mW ZEN and ZAN = 318.4 g/mol, mW α-ZEL and β-ZEL = 320.4 g/mol). ^5^ One participant had a major outlying value of 2.26 μg OTA/L, which led to a large SD. ^6^ LOD and LOQ values of each mycotoxin are reported in Section 4.3.3.

**Table 3:** Average, median and 95th percentile of DON EDIs for participants (*n* = 36).

	EDI (μg/kg bw/day)
**Average ± SD**	0.257 ± 0.127
**Median**	0.233
**95th percentile**	0.476
**Range**	0.0695–0.654

DON: deoxynivalenol; EDI: estimated daily intake; SD: standard deviation.

**Table 4:** Average, median and 95th percentile EDIs for ZEN and its metabolites, α-ZEL, β-ZEL and ZAN in participants (*n* = 36).

	EDI (μg/kg bw/day)	
	Worst-Case (FUE = 0.094)	Best-Case (FUE = 0.368)
**Average ± SD**	0.0367 ± 0.0184	0.00937 ± 0.00469
**Median**	0.0331	0.00846
**95th percentile**	0.0661	0.0169
**Range**	0.0156–0.0790	0.00399–0.0202

EDI: estimated daily intake; FUE: urinary excretion fraction; SD: standard deviation; ZAN: zearalanone; ZEN: zearalenone; α-ZEL: α-zearalenol; β-ZEL: β-zearalenol.

**Table 5:** Average, median and 95th percentile OTA EDIs for participants (*n* = 36).

	EDI (ng/kg bw/day)
**Average ± SD**	0.810 ± 1.48
**Median**	0.550
**95th percentile**	1.02
**Range**	0.197–9.36

EDI: estimated daily intake; OTA: ochratoxin A; SD: standard deviation.

**Table 6:** Recovery, repeatability and LOD/LOQ values of measured mycotoxins.

Mycotoxin	Mean Recovery ^1^ (%)	Repeatability (%)	LOD/LOQ (μg/L)
**AFM1**	111	9.6	0.002/0.005
**DON**	104	1.9	0.07/0.20
**DOM-1**	107	2.8	0.07/0.20
**ZEN**	88	14.5	0.005/0.01
**α-ZEL**	91	12.1	0.025/0.05
**β-ZEL**	102	25.7	0.025/0.05
**ZAN**	116	15.2	0.013/0.025
**FB1**	119	14.3	0.01/0.01
**OTA**	100	4.8	0.05/0.05

AFM1: aflatoxin M1; DOM-1: de-epoxy-deoxynivalenol; DON: deoxynivalenol; FB1: fumonisin B1; LOD: limit of detection; LOQ: limit of quantification; OTA: ochratoxin A; ZAN: zearalanone; ZEN: zearalenone; α-ZEL: α-zearalenol; β-ZEL: β-zearalenol. ^1^ Recovery following spiking at LOQ values for each mycotoxin.

## Data Availability

Data can be made available on request.

## Supplementary Materials

The following supporting information can be downloaded at: https://www.mdpi.com/article/10.3390/toxins16060278/s1. Table S1: LC-MS/MS specifications.
